# The secretome of liver X receptor agonist‐treated early outgrowth cells decreases atherosclerosis in *Ldlr*−/− mice

**DOI:** 10.1002/sctm.19-0390

**Published:** 2020-11-24

**Authors:** Adil Rasheed, Sarah A. Shawky, Ricky Tsai, Richard G. Jung, Trevor Simard, Michael F. Saikali, Benjamin Hibbert, Katey J. Rayner, Carolyn L. Cummins

**Affiliations:** ^1^ Department of Pharmaceutical Sciences, Leslie Dan Faculty of Pharmacy University of Toronto Toronto Ontario Canada; ^2^ Capital Research Group University of Ottawa Heart Institute Ottawa Ontario Canada; ^3^ Department of Cellular and Molecular Medicine, Faculty of Medicine University of Ottawa Ottawa Ontario Canada; ^4^ University of Ottawa Heart Institute Ottawa Ontario Canada; ^5^ Division of Cardiology University of Ottawa Heart Institute Ottawa Ontario Canada; ^6^ Department of Biochemistry, Microbiology and Immunology University of Ottawa Ottawa Ontario Canada; ^7^ Banting and Best Diabetes Centre Toronto Ontario Canada; ^8^ The Heart and Stroke Richard Lewar Centre of Excellence in Cardiovascular Research Toronto Ontario Canada

**Keywords:** atherosclerosis, autologous cell therapy, early outgrowth cells, liver X receptor, secreted factors

## Abstract

Endothelial progenitor cells (EPCs) promote the maintenance of the endothelium by secreting vasoreparative factors. A population of EPCs known as early outgrowth cells (EOCs) is being investigated as novel cell‐based therapies for the treatment of cardiovascular disease. We previously demonstrated that the absence of liver X receptors (LXRs) is detrimental to the formation and function of EOCs under hypercholesterolemic conditions. Here, we investigate whether LXR activation in EOCs is beneficial for the treatment of atherosclerosis. EOCs were differentiated from the bone marrow of wild‐type (WT) and LXR‐knockout (*Lxrαβ*−/−) mice in the presence of vehicle or LXR agonist (GW3965). WT EOCs treated with GW3965 throughout differentiation showed reduced mRNA expression of endothelial lineage markers (*Cd144*, *Vegfr2*) compared with WT vehicle and *Lxrαβ*−/− EOCs. GW3965‐treated EOCs produced secreted factors that reduced monocyte adhesion to activated endothelial cells in culture. When injected into atherosclerosis‐prone *Ldlr*−/− mice, GW3965‐treated EOCs, or their corresponding conditioned media (CM) were both able to reduce aortic sinus plaque burden compared with controls. Furthermore, when human EOCs (obtained from patients with established CAD) were treated with GW3965 and the CM applied to endothelial cells, monocyte adhesion was decreased, indicating that our results in mice could be translated to patients. Ex vivo LXR agonist treatment of EOCs therefore produces a secretome that decreases early atherosclerosis in *Ldlr*−/− mice, and additionally, CM from human EOCs significantly inhibits monocyte to endothelial adhesion. Thus, active factor(s) within the GW3965‐treated EOC secretome may have the potential to be useful for the treatment of atherosclerosis.

## Graphical abstract

Bone marrow cells differentiated with liver x receptors agonist (GW) produce a population of early outgrowth cells (EOCs) with an alternative secretome, capable of decreasing monocyte‐endothelial adhesion in culture, and reducing atherosclerosis in vivo. GW‐treated EOCs derived from peripheral blood mononuclear cells (MNCs) of patients with coronary artery disease also produced a secretome that reduced monocyte‐endothelial adhesion in culture.

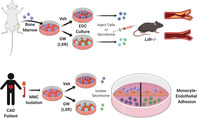

**Abbreviations**
CAD: coronary artery disease
CM: conditioned media
EndMT: endothelial‐to‐mesenchymal transition
EOC: early outgrowth cell
EPC: endothelial progenitor cell
HUVEC: human umbilical vein endothelial cell
LXR: liver X receptor
WT: wild type

**Significance statement**

Patient‐derived early outgrowth cells (EOCs) are being investigated as a potential treatment for vascular diseases, including pulmonary hypertension and myocardial infarction. The data of this study demonstrate that liver X receptor (LXR) activation of mouse EOCs causes the secretion of factors that reduce binding of inflammatory cells to endothelial cells and ultimately decrease the development of atherosclerosis. This study also shows that LXR agonist‐treated EOCs derived from human coronary artery disease patients recapitulate these results in cell culture. Therefore, this study suggests that treatment with autologous LXR agonist‐treated EOCs and/or the isolation of the beneficial factors that are secreted may help decrease the progression of atherosclerosis.

## 1 INTRODUCTION

Cardiovascular disease remains the leading cause of death worldwide.^1^ Atherosclerosis is a vascular complication arising from cardiovascular disease and is typically diagnosed in its later stages, after established lipid‐laden plaques have deposited in the aorta. Plaque development is initiated by coordinate dysfunction to various vessel wall cell types, including endothelial and vascular smooth muscle cells, as well as hematopoietic cell types.^2^ Prior to plaque deposition, the aortic endothelium undergoes pathological activation resulting from systemic inflammation and elevations in plasma lipoproteins. During endothelial activation, selectins and adhesion molecules are upregulated, which facilitate the binding of monocytes to the endothelium. Adherent monocytes then traverse the endothelium to the intima, and differentiate into macrophages which engulf modified lipoproteins to promote the plaque development characteristic of established atherosclerosis.^3^

The liver X receptors, LXRα (*Nr1h3*) and LXRβ (*Nr1h2*), belong to the nuclear receptor superfamily of transcription factors.^4^, ^5^ Activation of the LXRs induces the gene expression of the cholesterol efflux transporters, *Abca1* and *Abcg1*; and represses pro‐inflammatory gene expression, including *Mcp‐1*, *Tnfα*, and *Il‐1β*.^6^, ^7^, ^8^ These roles of LXRs have been particularly well characterized in macrophages, in part through bone marrow transplant experiments that support an anti‐atherogenic role for LXRs.^9^, ^10^, ^11^, ^12^, ^13^, ^14^ However, recent bone marrow transplantation studies using bone marrow deficient in LXR target genes have demonstrated that LXRs may elicit their anti‐atherogenic roles in other bone marrow‐derived cell types apart from monocytes/macrophages.^14^, ^15^, ^16^, ^17^ In the bone marrow, hematopoietic stem cells (HSCs) can differentiate into myeloid progenitors, which produce the monocytes/macrophages known to contribute directly to plaque progression.^3^, ^18^ HSCs, however, can also differentiate into other cell types including endothelial progenitor cells (EPCs).^19^, ^20^

EPCs were initially described in a report by Asahara et al in 1997 as peripheral blood cells that differentiate into endothelial‐like cells and contribute to vascular repair by direct incorporation.^19^ Many studies have since demonstrated that patients with diabetes or cardiovascular disease have dysfunction in EPC numbers and function.^21^, ^22^, ^23^ Over the past 20 years, EPC function has largely been characterized using ex vivo cultures, which yield two cell populations: early outgrowth cells (EOCs; 7‐10 days in culture) and late outgrowth endothelial cells (14‐21 days in culture). EOCs participate in endothelial repair through the secretion of factors, whereas late outgrowth cells differentiate to endothelial‐like cells and promote vascular homeostasis through direct incorporation.^24^, ^25^, ^26^, ^27^ Consistent with observations made by Asahara and colleagues, EPC studies were initially focused on their endothelial‐like phenotype and how they facilitate neovascularization by incorporation into the damaged vessel.^28^, ^29^, ^30^, ^31^, ^32^, ^33^ However, studies have since shown inconsistent engraftment in vivo, suggesting that endothelial repair by EPCs occurs via an indirect mechanism.^26^, ^34^, ^35^, ^36^, ^37^ Given these discrepancies, attention has shifted to the EOC subpopulation that supports endothelial repair by the secretion of vasoreparative factors, which has spurred a new generation of clinical trials^38^, ^39^ and applications in regenerative medicine, such as those reviewed by Chong et al.^40^

Using *Lxrαβ*−/− double knockout mice fed a hypercholesterolemic diet, we previously demonstrated that LXRs are essential for preventing cholesterol‐induced defects in EOCs.^41^ Elevated cholesterol content, only in the absence of LXRs, altered EOC differentiation and the resulting secretome, which increased monocyte adhesion to treated endothelial cells in vitro. As a result of these studies, here we set out to determine: (a) whether pharmacologic activation of LXRs could beneficially influence EOC differentiation and secretome function and (b) if ex vivo pharmacological activation of LXRs in EOCs could prevent the development of atherosclerosis in vivo. Herein, we present data that show pharmacological activation of LXRs reduces the expression of endothelial lineage markers in wild‐type (WT) EOCs, and alters the EOC secretome in a manner that inhibits monocyte adhesion to treated endothelial cells. Tail vein administration of either LXR agonist‐treated WT EOCs or their conditioned media (CM) to *Ldlr*−/− mice attenuated lesion development in the aortic sinus compared to their respective controls. Furthermore, we demonstrate the translational potential of LXR agonist‐treatment of EOCs using EOCs derived from patients with established coronary artery disease (CAD). The studies presented here suggest that activation of LXRs in EOCs ex vivo may therefore provide a new avenue for therapeutic intervention in atherosclerosis.

## 2 MATERIALS AND METHODS

### 2.1 Mice

All animal procedures were approved by the Institutional Animal Care and Use Committee at the University of Toronto. WT and LXR double knockout (*Lxrαβ*−/−) male mice were backcrossed more than 10 generations on a C57Bl/6 background and maintained on a chow diet (#2016 Harlan Teklad, Mississauga, Ontario, Canada). *Ldlr*‐knockout male mice (*Ldlr*−/−; B6.129S7‐Ldlr^*tm1Her*^/J, Stock #002207) on a C57Bl6/J background were purchased from Jackson laboratories (Bar Harbor, Maine). All mice were housed in a temperature and light‐controlled environment. Mice were sacrificed between 9 am and 11 am by cervical dislocation or exsanguination under isoflurane anesthesia.

### 2.2 Bone marrow harvesting and EOC culture

EOCs were cultured from the bone marrow of the tibiae and femurs of WT and *Lxrαβ*−/− mice. The bone marrow cells were seeded on human‐fibronectin coated (Sigma‐Aldrich, Oakville, Ontario, Canada) tissue culture dishes and differentiated for 7 days to EOCs in endothelial basal media supplemented with growth factors/cytokines (EGM‐2 Bullet Kit; Lonza, Walkersville, Maryland) at 37°C with 5% CO_2_. The media was changed every other day. EOCs were cultured in the presence of vehicle (ethanol) or LXR agonist (1 μM GW3965; Tocris, Minneapolis, Minnesota) throughout the 7‐day differentiation protocol.

### 2.3 RNA isolation, cDNA synthesis, and real‐time QPCR

RNA isolation, cDNA synthesis, and QPCR were performed as previously described.^41^ Primer sequences are listed in Supporting Information Table S1 (mouse) and Supporting Information Table S2 (human).

### 2.4 CAD patient recruitment

Patients who underwent coronary angiography gave written informed consent for blood collection. This study was approved by the Ottawa Health Science Network Research Ethics Board (OHSN‐REB, Protocol #: 20160516‐01H). This study conforms to the 1975 Declaration of Helsinki for the use of human blood. The University of Ottawa Heart Institute serves 1.2 million people with all cardiac catheterization procedures prospectively indexed in the Cardiovascular and Percutaneous Clinical Trials (CAPITAL) revascularization registry along with baseline investigations and medications.^42^, ^43^ From August 2018 to March 2019, blood samples were collected from 21 patients in EDTA tubes (Becton Dickinson, Franklin Lakes, New Jersey) at the time of coronary angiography. CAD was confirmed by coronary angiography and defined as an epicardial stenosis ≥50%. The presence of type 2 diabetes was defined as hemoglobin A1c (HbA1c) levels ≥6.5% or a prior diagnosis or medical therapy for diabetes. Patient characteristics are provided in Table 1.

**TABLE 1:** Characteristics of coronary artery disease patients from which EOCs were derived

	EOC (n = 21)	
	Number or mean ± SD	Proportion (%)
Age, years	68 ± 13	
Sex (male)	15	71.4
HTN	12	57.1
DM		
Type I	1	4.8
Type II ‐ oral agents	4	19.1
Type II ‐ insulin	2	9.5
Smoker		
Never	16	76.2
Remote (quit >1 month ago)	3	14.3
Active	2	9.5
Dyslipidemia	12	57.1
Family history of CAD	3	14.3
ACS	7	33.3
HF/LV dysfunction	2	9.5
Prior history		
MI	4	19.1
PCI	3	14.3
CVA	1	4.8
CABG	1	4.8
Medications		
ASA	20	95.2
P2Y12	18	85.7
Beta blocker	10	47.6
ACEi/ARB	14	66.7
Statin	19	90.5
Biochemistry		
WBC (10^9^ cells/L)	7 ± 2	
HGB (g/L)	134 ± 26	
PLT (10^9^ cells/L)	225 ± 52	
HbA1c (%) (n = 16)	6.1 ± 0.8	
Cholesterol (mM) (n = 7)	4 ± 1	
LDL (mM) (n = 7)	2 ± 1	
Creatinine (μM)	92 ± 65	

Abbreviations: ACE, angiotensin‐converting enzyme; ACS, acute coronary syndrome; ARB, angiotensin receptor blocker; ASA, acetylsalicylic acid; CABG, coronary artery bypass graft; CVA, cerebrovascular accident; DM, diabetes mellitus; HF/LV, heart failure/left ventricular; HGB, hemoglobin; HTN, hypertension; LDL, low‐density lipoprotein; MI, myocardial infarction; PCI, percutaneous coronary intervention; PLT, platelet; WBC, white blood cell.

### 2.5 Human EOC culture

Peripheral blood mononuclear cells were isolated by density centrifugation using Ficoll‐Paque Plus (GE Healthcare, Marlborough, Massachusetts). The cells were differentiated to EOCs as described above. For each patient, the mononuclear cells were differentiated to EOCs in both vehicle and 1 μM GW3965.

### 2.6 CM collection

After 7 days of differentiation, EOCs were washed twice with cold PBS and incubated in factor‐ and GW3965‐free media (EBM‐2; Lonza) for 30 minutes at 37°C and 5% CO_2_. The media was discarded and the cells were replenished with fresh EBM‐2 media. The CM was then collected after 24 hours, centrifuged at 700*g* for 5 minutes to remove any cells, and syringe filtered (0.22 μm). The CM was frozen at −80°C until use. The number of EOCs from each treatment group was unchanged after differentiation (data not shown).

### 2.7 Cell lines

Human umbilical vein endothelial cells (HUVEC) and media (EGM‐2 Bullet Kit) were purchased from Lonza. THP‐1 monocytes were received as a gift from Dr. Myron I. Cybulsky (University of Toronto) and were maintained in RPMI 1640 with l‐glutamine (Sigma‐Aldrich) supplemented with 10% FBS and 0.05 mM β‐mercaptoethanol (Sigma‐Aldrich). All cell lines were used between passages 4 and 6.

### 2.8 Monocyte‐endothelial adhesion assay

HUVECs were seeded in 12‐well plates on sterilized circular glass coverslips (Thermo Fisher Scientific, Hampton, New Hampshire) and treated at 60% confluence with 20% (mouse) or 30% (human) EOC CM in EGM‐2 media for 20 hours, followed by a 4‐hour cotreatment with EOC CM and 10 ng/mL TNFα (Life Technologies). After treatment, HUVECs were coincubated with 10^5^ CMFDA (Life Technologies)‐labeled THP‐1 monocytes per well for 90 minutes. After coculture, the nonadherent THP‐1 cells were washed off and the cells were fixed using 4% paraformaldehyde. Coverslips were mounted onto microscope slides using Vectashield (Vector Labs, Burlingame, California) or Dako Fluorescence (Agilent Technologies, Santa Clara, California) and images were acquired on a laser confocal LSM700 or LSM880 microscope operated by Zen software (Zeiss, Toronto, Ontario, Canada).

### 2.9 Splenectomy

A cohort of *Ldlr*−/− mice were splenectomized at 4 weeks of age. The abdominal cavity was opened, and the spleen was removed by cauterizing the splenic arteries. The abdominal cavity and skin were closed using 4‐0 gauge synthetic absorbable suture. The mice were then treated with ketoprofen (s.i.d.) and buprenorphine (b.i.d.) for 3 days postsurgery and allowed to recover 4 weeks prior to the initiation of treatments and diet.

### 2.10 Preparation of EOCs and concentrated CM for in vivo studies

To generate EOCs and their CM, bone marrow from the femurs and tibiae of two mice were seeded in one 100 mm plate. Day 7 EOCs were detached using Accutase Enzyme Detachment Buffer (eBioscience, San Diego, California) washed once, and resuspended in saline for injections. Approximately 10^7^ EOCs were collected at day 7, irrespective of ligand treatment. CM was concentrated 10‐fold using a <3 kDa Amicon centrifuge filtration column (Millipore, Etobicoke, Ontario, Canada) and dialyzed twice in saline prior to injection.

### 2.11 Treatment and sacrifice of Ldlr−/− mice

Starting at 8 weeks of age, *Ldlr*−/− mice were fed a Western style diet (#88137 Harlan Teklad) for 8 weeks. The splenectomized cohort of the *Ldlr*−/− mice were injected via the tail vein with 10^6^ EOCs from WT C57Bl/6 mice in 100 μL of saline. The nonsplenectomized *Ldlr*−/− mice received 100 μL of the 10‐fold concentrated CM. Treatments were administered every 2 weeks throughout the Western diet feeding period. Complete blood counts were performed on peripheral blood collected at sacrifice on a VetScan HM5 (Abaxis, Union City, California). Whole blood obtained at sacrifice was centrifuged at 500*g* for 20 minutes at 4°C for the separation of plasma, which was aliquoted and frozen at −80°C until analysis. Plasma cholesterol was determined by enzymatic assay using the Cholesterol Infinity Kit (Thermo Fisher Scientific) and IL‐6 quantified by ELISA (Thermo Fisher Scientific) as per the manufacturer's recommendations.

### 2.12 Histological analysis

Aortic sinuses were cryosectioned using a Leica CM 3050S cryostat (Concord, Ontario, Canada) at a 10 μm thickness and placed on Superfrost Plus microscope slides (Thermo Fisher Scientific). For lesion quantification, the sections were stained with hematoxylin and eosin (Sigma‐Aldrich). For immunohistochemical analyses, the sections were incubated with the primary antibodies VCAM1 (CD106, BD Biosciences, 1:10) or CD45 (BD Biosciences, 1:20) overnight at 4°C followed by the biotinylated anti‐rat IgG secondary antibody (Vector Laboratories, 1:100). The sections were washed and then incubated with the ABC kit (Vector Laboratories) for 30 minutes following manufacturer's recommendations, followed by incubation with 3,3′‐diaminobenzidine tetrahydrochloride (DAB, Sigma‐Aldrich). The sections were then counterstained with hematoxylin. Images were acquired using a Mirax Scan slide scanner (Zeiss) at ×20 magnification. Section images were analyzed by a scientist blinded to the treatment conditions using ImageJ (NIH, Bethesda, Maryland). Sections for which all three valves were present were quantified and averaged for each mouse. Lesion area was manually traced for each section, quantified in μm^2^ and reported as a percentage of the total vessel area to account for differences in total aortic sinus cross‐sectional area between mice. The endothelial surface was manually traced with a 20 μm thick line and VCAM1‐positive staining was reported as a percentage of this line area. CD45‐positive staining was quantified only in the lesions and reported as a percentage of total lesion area.

### 2.13 Proteomic analysis

EOC cell pellets were lysed with RIPA buffer and 100 μg of protein was digested using the FASP method^44^ on 30 kDa spin filters (Millipore). The eluted peptides were acidified and desalted using in‐house made C18 pipette tips (10 μg capacity). Analysis was performed on an Easy nLC‐1200 coupled to a ThermoQExactive HF mass spectrometer (Thermo Fisher Scientific) operating in a top 20 mode. The mobile phase was composed of Buffer A (0.1% formic acid) and Buffer B (0.1% formic acid in 80% acetonitrile). Peptides were separated using a PepMap RSLC C18 2 μm, 75 μm × 50 cm column and a PepMap 100 C18 3 μm, 75 μm × 2 cm precolumn with a 2 hour gradient of 5% to 40% Buffer B. Data were analyzed using MaxQuant (v1.6.10.43)^45^ and Perseus.^46^

### 2.14 Statistical analysis

Statistical analyses were performed using GraphPad Prism (version 6; GraphPad Software Inc., La Jolla, California). Data are presented as mean ± SD or SEM, as indicated in the figure legends. Data with n ≥8 were subjected to the D'Agostino and Pearson omnibus normality test and found to conform to a normal distribution. One‐way ANOVA, followed by the Holm's Sidak's post hoc test, was used for comparison between more than two groups. For the human EOC study, no sample size calculation was performed as the analysis was considered exploratory. A parametric paired two‐tailed *t* test was used to compare treatment groups in the human EOC studies. A value of *P* < .05 was considered to be statistically significant.

## 3 RESULTS

### 3.1 GW3965 alters differentiation of EOCs to the endothelial lineage

**FIGURE 1:**
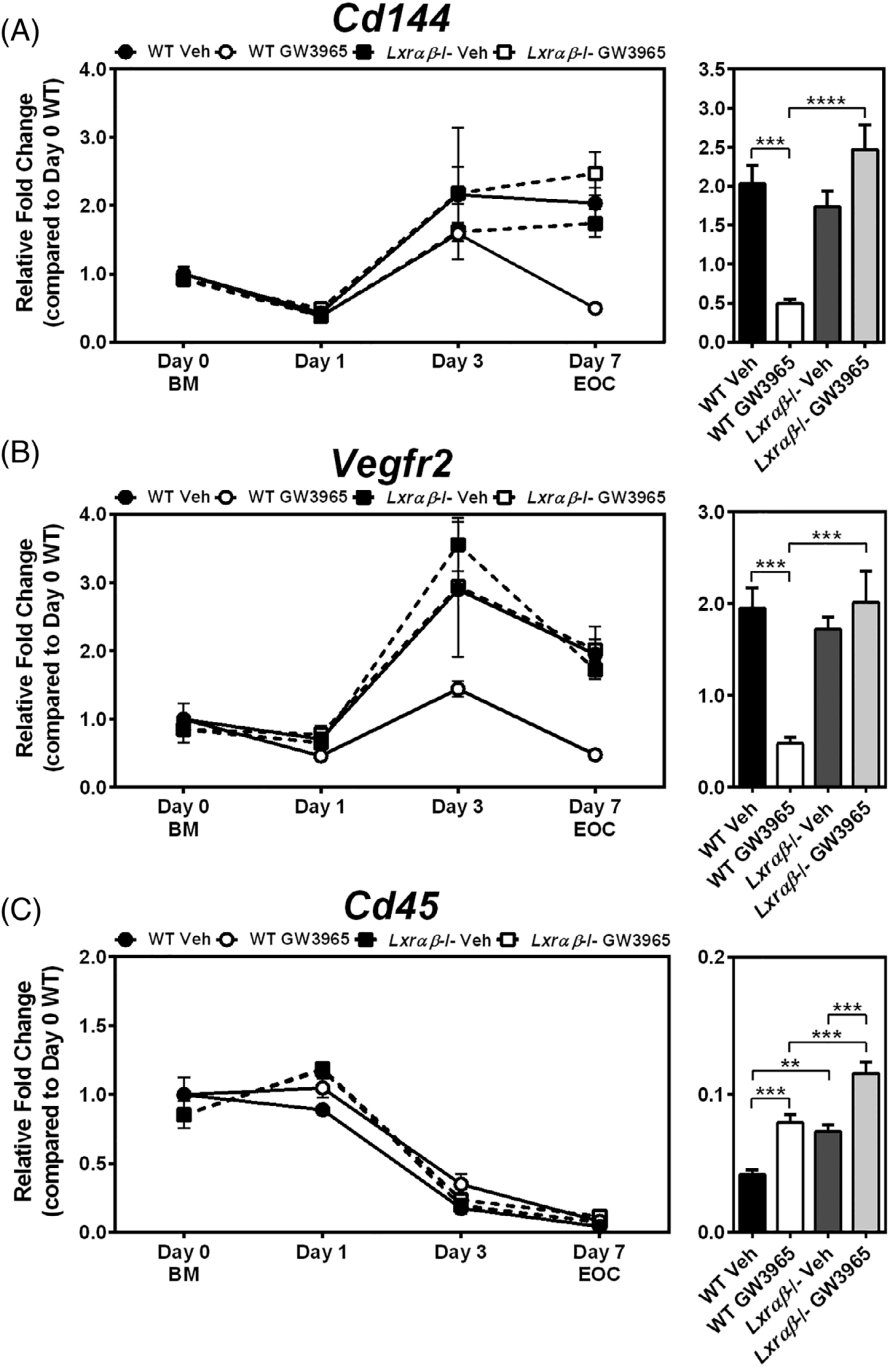
Wild‐type EOCs treated with LXR agonist have lower expression of endothelial markers after differentiation. A‐C, Bone marrow (BM; Day 0) from 12‐week old WT and *Lxrαβ*−/− mice were differentiated for 7 days in the presence of LXR agonist (1 μM GW3965). Gene expression was performed for the endothelial lineage markers (A) *Cd144* and (B) *Vegfr2*, as well as (C) the leukocyte marker *Cd45*. Time course analyses are shown in the left panels, and day 7 EOC expression levels in the right panel. n = 5‐6 per group. Data represent the mean ± SEM. **P < .01, ***P < .001, ****P < .0001

We previously demonstrated using a loss‐of‐function model that LXRs play an important role in mitigating the negative effects of cholesterol, either from Western diet feeding or addition of exogenous cholesterol, on EOC differentiation.^41^ Here, we set out to determine whether LXR activation would have an opposing role on EOC differentiation. Treatment with LXR agonist (1 μM GW3965) throughout EOC differentiation decreased the expression of the endothelial lineage markers *Cd144* and *Vegfr2* both by 76% in day 7 EOCs derived from WT mice, but not *Lxrαβ*−/− mice (*P* < .001; Figure 1A,B). Interestingly, GW3965 treatment resulted in a varied time course profile for these endothelial genes. WT cells differentiated in the presence of GW3965 showed similar expression of *Cd144* compared to vehicle treated cells up to day 3. However, after day 3, *Cd144* expression was decreased in the WT GW3965‐treated EOCs compared to vehicle treatment, suggesting a de‐differentiation program (Figure 1A). On the other hand, the increase in *Vegfr2* expression normally observed with differentiation was prevented in WT cells treated with GW3965 (Figure 1B).

We next examined the expression of leukocyte and myeloid markers, which are expected to decrease during differentiation. Time course analysis did reveal potent reductions of the pan leukocyte marker *Cd45* by day 3 of differentiation, and while GW3965 treatment appeared to delay this decrease at day 7 in EOCs, this effect was not LXR‐dependent (Figure 1C). Furthermore, at day 7, expression of the myeloid marker *Cd11b* was not altered by GW3965 in WT EOCs (Supporting Information Figure S1A). During atherosclerosis, endothelial to mesenchymal transition (EndMT) induced by oxidative stress, TGFβ, and other stressors characteristic of the atherosclerotic milieu, can cause endothelial cells to acquire a more fibrotic phenotype and express mesenchymal markers such as *Tgfβ* and *Fsp‐1*.^47^, ^48^ Notably, while LXR activation decreased EOC expression of endothelial lineage markers (*Cd144* and *Vegfr2*), expression of these mesenchymal markers in the GW3965‐treated WT EOCs was not increased (Supporting Information Figure S1B,C), suggesting that these EOCs were not undergoing EndMT. These data indicate that while GW3965 treatment to WT cells decreased differentiation to the endothelial lineage, expression of the leukocyte/myeloid markers *Cd45* and *Cd11b* were not increased, suggesting that agonist treatment did not prevent differentiation or induce de‐differentiation, but rather produced an altered population of EOCs.

To assess the effect of GW3965 at the protein level we performed shotgun proteomics on the EOCs after 1 day or 9 days of differentiation. While CD144 and VEGFR2 proteins were not detectable, we did observe increased EOC expression of another classic endothelial marker, von Willebrand factor (vWF) during differentiation (day 1 vs day 9). Similar to the trends observed for other endothelial markers at the gene expression level, EOC differentiation in the presence of GW3965 resulted in decreased levels of vWF compared to vehicle (Supporting Information Figure S1D). Unsupervised clustering of the data demonstrated that each of the three treatment groups clustered separately, supporting the idea that the GW3965 treatment creates a distinct EOC sub‐population (Supporting Information Figure S1E).

### 3.2 Secretome of GW3965‐treated EOCs reduced monocyte endothelial adhesion

**FIGURE 2:**
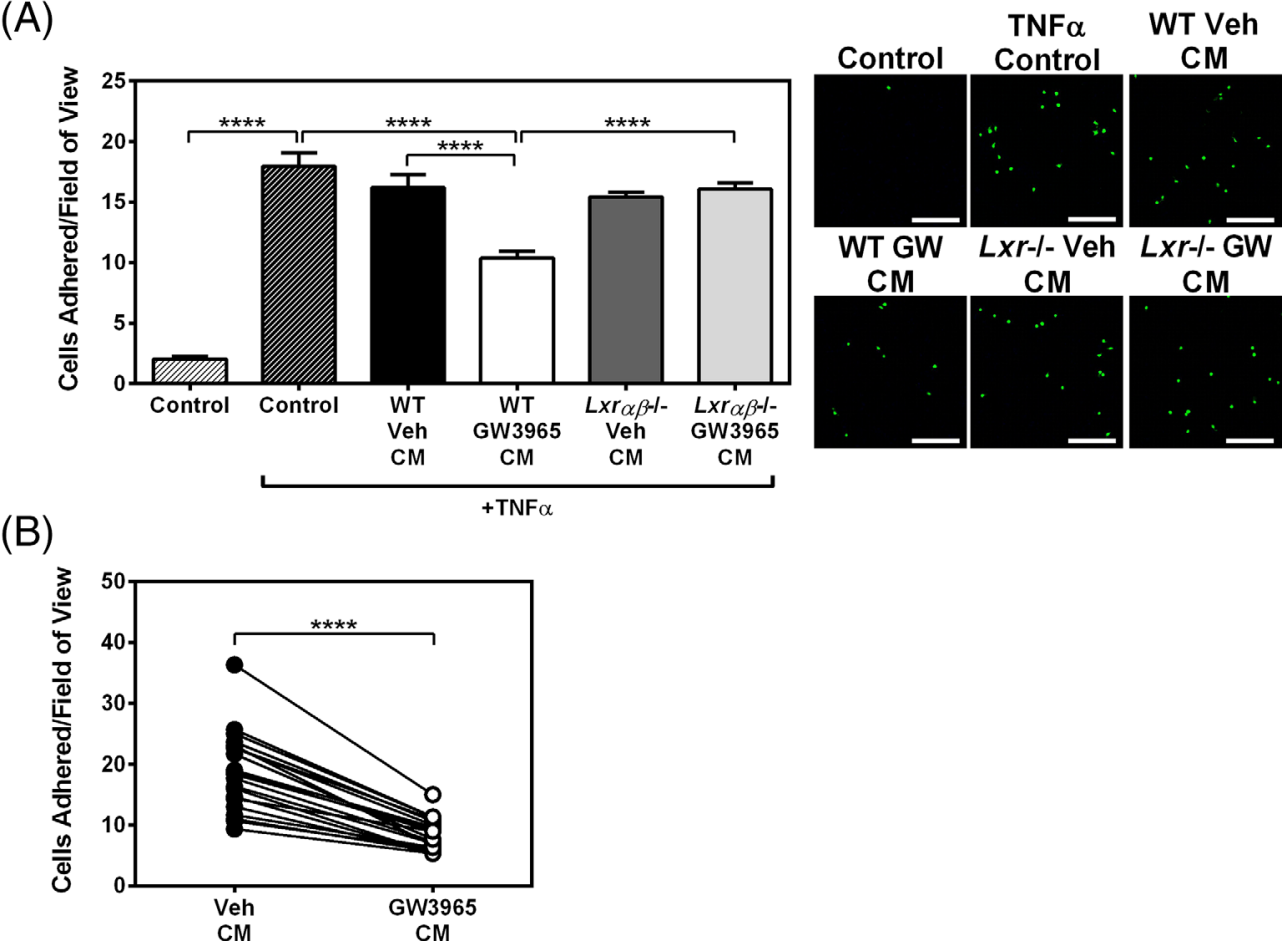
Secreted factors from GW3965‐treated mouse and patient‐derived EOCs reduce monocyte adherence to activated endothelial cells. A,B, Conditioned media from GW3965‐treated EOCs were harvested and applied to HUVECs treated with TNFα. Monocyte adhesion to treated HUVECs was quantified after incubation with conditioned media derived from (A) WT and *Lxrαβ*−/− mouse EOCs (n = 8 per group) and (B) EOCs derived from patients with coronary artery disease (n = 21 patients). Scale bar = 200 μm. Data represent the mean ± SEM. ****P < .0001

To investigate the impact of LXR‐activated EOCs during the early stages of atherosclerosis, we used the in vitro monocyte‐endothelium adhesion assay. HUVECs activated with TNFα were incubated with CM from vehicle‐ or GW3965‐treated EOCs prior to the addition of fluorescently labeled THP‐1 monocytes. As expected, TNFα‐treatment increased monocyte adhesion to HUVECs by 8.8‐fold (*P* < .0001; Figure 2A). Treatment of HUVECs with CM from GW3965‐treated WT EOCs (WT GW CM) decreased the adhesion of THP‐1 cells by 36% compared to WT Veh CM (*P* < .0001; Figure 2A). In contrast, CM from GW3965‐treated *Lxrαβ*−/− EOCs (*Lxr*−/− GW CM) had no effect on TNFα‐induced monocyte adherence. These results indicate that LXR activation altered the EOC population, which in turn produced a secretome that decreased monocyte adhesion to activated endothelial cells.

During atherosclerosis, endothelial cells upregulate the expression of selectins, which facilitate leukocyte rolling, and adhesion molecules that enhance leukocyte adhesion to the aortic endothelium.^3^ To determine whether WT GW CM decreased monocyte adhesion by altering the expression of adhesion molecules via signaling to the HUVECs, we performed gene expression analysis of the HUVECs after incubation with EOC CM. We observed that while the addition of the pro‐inflammatory stimulus TNFα did induce the expression of the adhesion molecules *VCAM1* and *ICAM1*, as well as *SELE*, there was no change in any of these markers of endothelial activation when co‐treated with WT GW CM compared the WT Veh CM (Supporting Information Figure S2A). To establish whether the CM was required to “prime” the endothelial cells prior to TNFα administration, we tested whether CM administered only in the pre‐TNFα period (20 hours) and then washed out; or only during TNFα treatment (ie, for 4 hours concurrent with TNFα) would be sufficient to elicit the beneficial effect to decrease monocyte‐endothelial binding. Interestingly, CM was effective at decreasing monocyte adhesion under both conditions and the effect was most pronounced when included both before and during TNFα (Supporting Information Figure S2D). These data suggest that components in the secretome can both neutralize the effect of TNFα and prime the endothelial cells to protect them from TNFα‐mediated activation. The detailed mechanisms by which WT GW CM alters monocyte‐HUVEC adhesion, however, remain elusive.

We next set out to determine whether the therapeutic effects of the GW3965 on the EOC secretome could be translated to patients with CAD. For this, we obtained peripheral blood samples from a CAD cohort with characteristics described in Table 1. Cells were differentiated to EOCs in the presence of vehicle or 1 μM GW3965 and the CM was collected at day 7. Similar to EOCs derived from mouse bone marrow, monocyte adhesion was decreased by 54% when HUVECs were treated with the CM from GW3965‐treated EOCs from the CAD patients compared to vehicle treatment (*P* < .0001; Figure 2B). This therefore demonstrated that GW3965 improved EOC function from CAD patients to a similar extent as that observed in murine cells from WT mice.

### 3.3 Administration of EOCs and their secreted factors reduce plaque size during atherogenesis

**FIGURE 3:**
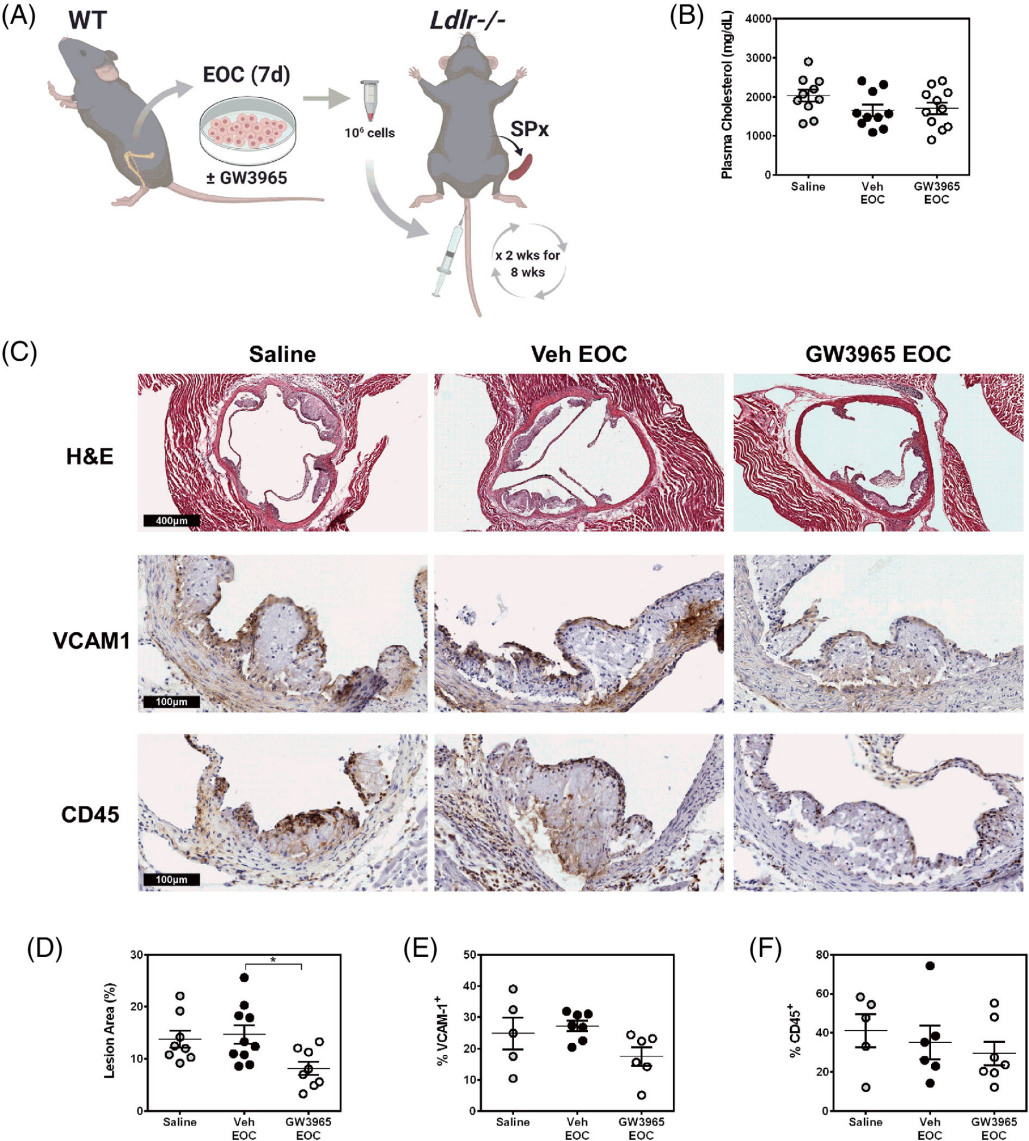
LXR agonist‐treated EOCs reduce plaque area in the aortic sinus. A, EOCs derived from WT mice were differentiated in the presence of GW3965 and administered to splenectomized *Ldlr*−/− mice bi‐weekly over an 8‐week period. B, Plasma cholesterol levels were quantified from the blood collected at sacrifice. C‐F, Histological analyses were performed on the aortic sinus sections for total lesion area (C, D) and percent positive areas for VCAM1 (C, E) and CD45 (C, F). n = 5‐11 mice per group. Data represent the mean ± SEM. *P < .05

Ex vivo cultured EOCs are currently undergoing clinical trials for the treatment of vascular complications arising from cardiovascular disease, such as pulmonary hypertension and acute myocardial infarction.^38^, ^39^, ^40^ In our study, we wanted to assess the therapeutic potential of WT GW3965‐treated EOCs and their secretome on the development of atherosclerosis using the atherogenic *Ldlr*‐knockout (*Ldlr*−/−) mouse model (Figure 3A). The *Ldlr*−/− mice receiving EOCs were splenectomized prior to initiation of the treatments to prevent sequestration of EOCs within the spleen.^49^ Plasma cholesterol levels were not changed with EOC administration (Figure 3B). Likewise, the number of circulating leukocytes were unchanged in *Ldlr*−/− mice with EOC administration compared to saline injected mice (Supporting Information Table S3). Histological analysis of the aortic sinus revealed a reduction in the luminal plaque coverage of the *Ldlr*−/− mice receiving the GW3965‐treated EOCs compared to those receiving the Veh‐treated EOCs (44%, *P* < .05) or saline (41%, n.s.) (Figure 3C). Despite these reductions in plaque area, there were no changes in endothelial activation (VCAM1) or leukocytes (CD45) within the plaque upon the administration of GW3965‐treated EOCs (Figure 3C).

**FIGURE 4:**
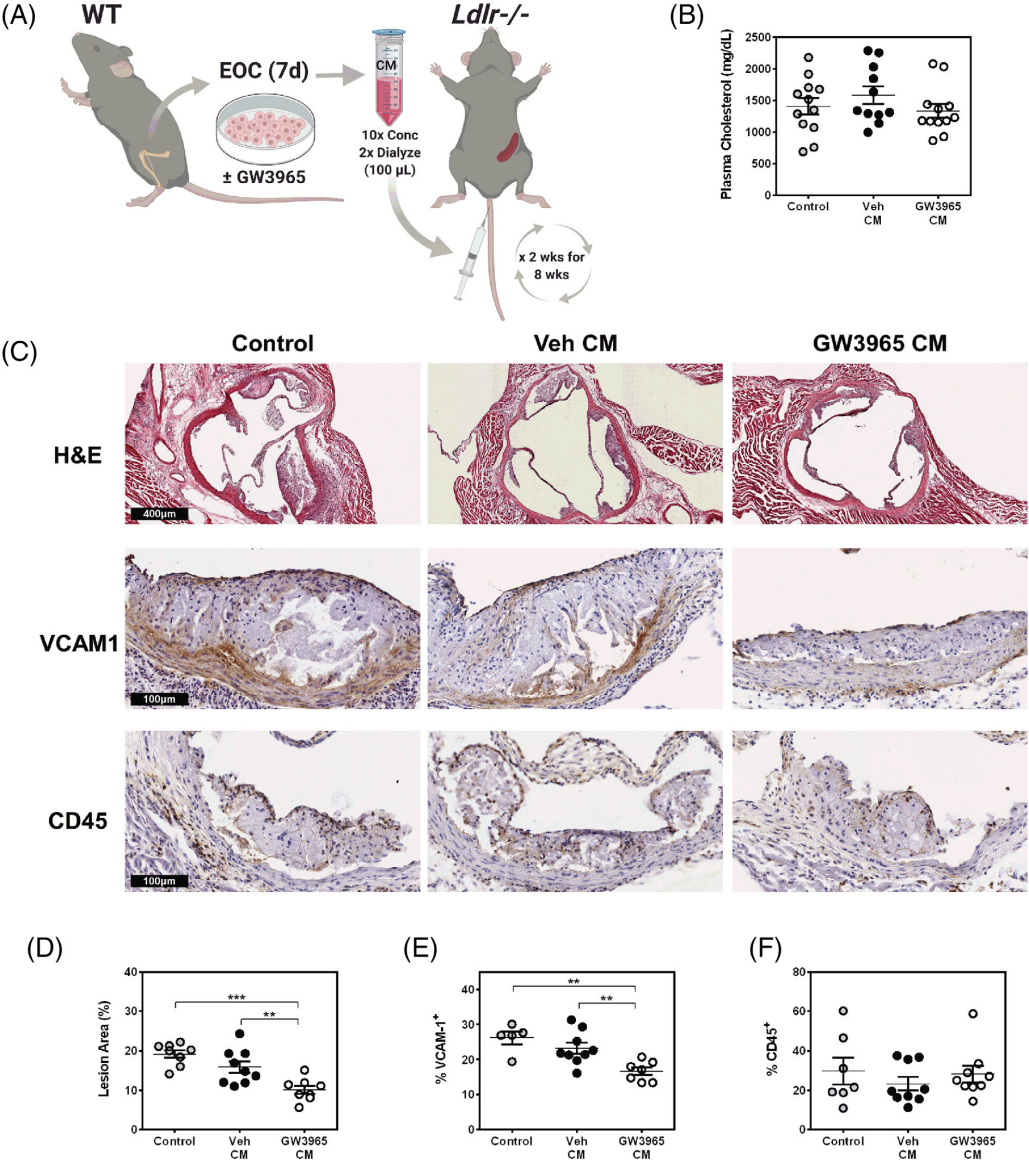
The secretome of GW3965‐treated EOCs decreases atherosclerotic plaque burden. A, The conditioned media from GW3965 treated EOCs were harvested and administered to *Ldlr*−/− mice bi‐weekly over 8 weeks. B, Plasma cholesterol levels were assessed in samples collected at sacrifice. C‐F, Histological analyses were performed on the aortic sinus sections for total lesion area (C, D) and percent positive areas for VCAM1 (C, E) and CD45 (C, F). n = 5‐12 mice per group. Data represent the mean ± SEM. **P < .01, ***P < .001

Since our in vitro results found a potent effect of the EOC secretome on limiting monocyte‐endothelial adhesion (Figure 2A), we administered CM from treated EOCs or control (unconditioned) media bi‐weekly to *Ldlr*−/− mice (with an intact spleen) to determine whether this alone would be sufficient to impact lesion formation (Figure 4A). We did not observe changes in plasma cholesterol upon injection of the CM (Figure 4B). Likewise, there was no effect on total blood counts of mice treated with CM, with the exception of mice receiving CM from GW3965‐treated EOCs in which monocytes were unexpectedly increased (Supporting Information Table S4). Remarkably, treatment with CM from GW3965‐treated EOCs reduced atherosclerotic plaques in the aortic sinus by 36% (*P* < .01) compared to Veh‐treated EOC CM and 47% (*P* < .001) compared to control unconditioned EBM‐2 media (Figure 4C). The CM from the GW3965‐treated EOCs significantly reduced VCAM1, the adhesion molecule responsible for leukocyte adhesion to the inflammatory endothelium during atherogenesis (Figure 4C). The proportions of CD45, a pan‐leukocyte marker, were not altered; indicating in part that the CM did not alter the content of the plaque but rather through the effects on the endothelium reduced total lesion area. No differences in plasma IL‐6 levels or spleen weights were found among the treatment groups (Supporting Information Figure S3), suggesting no overall effect on systemic inflammatory status. While the molecular component(s) contributing to these changes are not clear, these data demonstrate the therapeutic potential of the GW3965‐treated EOC secretome to limit the development of atherosclerosis in mice, with in vitro evidence that this response will be conserved in humans.

## 4 DISCUSSION

Atherosclerosis is a progressive disease that manifests over several decades. The gold standard treatment for atherosclerosis relies on lipid‐lowering, such as the administration of statin drugs and more recently, PCSK9 inhibitors.^50^ However, results from the Canakinumab Anti‐inflammatory Thrombosis Outcome Study (CANTOS) trial found that targeting the inflammatory axis can reduce cardiovascular‐related mortality, independent of lipid lowering.^50^, ^51^ These data, among others, emphasize the opportunity for the development of novel therapeutic avenues for the treatment of atherosclerosis that go beyond traditional lipid lowering modalities.

During the early stages of atherosclerosis, damage that occurs within the atherosclerotic environment overwhelms endogenous endothelial repair mechanisms, resulting in an overall pro‐inflammatory response that allows for an increase in endothelial expression of adhesion molecules that facilitate leukocyte binding.^3^ While the endothelium has the capability to repair itself, endothelial repair can also be supported by EOCs through the secretion of vasoreparative factors. We previously described a role for LXRs in mitigating the negative effects of high cholesterol‐induced defects on EOC differentiation and the secretome.^41^ Given the importance of EOCs in mediating endothelial repair via the secretome and the contributions of endothelial activation to the early pathogenesis of atherosclerosis, we tested whether pharmacologic activation of LXRs in EOCs produces a secretome that could reduce the progression of early stage atherosclerosis. Our data here show that LXR activation during EOC differentiation decreases the expression of endothelial lineage markers. This shift in differentiation qualitatively alters the secretome of the GW3965‐treated EOCs in a favorable manner, resulting in less monocyte‐endothelial adherence and an overall decrease in atherosclerosis. Along with our previously published data,^41^ this therefore provides a model whereby expression of endothelial markers is inversely associated with the vasoreparative benefit of the EOC secretome.

In contrast to the loss‐of‐function model, in which increased cholesterol in the *Lxrαβ*−/− EOCs correlated with increased expression of endothelial lineage markers^41^; the gain‐of‐function LXR model studied here demonstrated a decrease in these endothelial markers in the absence of changes in EOC cholesterol content (data not shown). These data suggest that there are separate mechanisms regulating EOC differentiation under the influence of cholesterol when LXRs are absent compared to when they are activated. Pharmacologic activation of LXRs throughout differentiation specifically decreased the expression of the endothelial lineage markers *Cd144* and *Vegfr2*. Time course analysis revealed that GW3965 treatment prevented the increase of *Vegfr2*, while *Cd144* expression decreased after day 3 of differentiation, compared to vehicle‐treated cells. Further experiments will be needed to confirm the mechanism by which EOC differentiation to the endothelial lineage is impacted by the activation of LXR, and how this occurs for each of these endothelial markers. These experiments will need to determine whether LXRs are directly binding to the DNA to inhibit endothelial marker expression or interact with other factors to inhibit transcriptional machinery at these individual loci.

We attempted to narrow down potential mechanisms by which GW3965‐treated EOC derived CM decreased monocyte to endothelial adhesion using gene expression analysis of HUVECs. We found no significant differences in marker genes associated with enhanced adhesion. While VCAM1 was not downregulated by treatment with the CM from GW3965‐treated EOCs in culture, administration of the CM to atherosclerosis‐prone mice did show a downregulation of endothelial VCAM1 in the aortic sinus, which was associated with a decrease in plaque area. The discrepancy between our in vitro and in vivo results could be due to exposure time (ie, in vitro the HUVEC cells were exposed to CM for only 24 hours vs in vivo CM was injected every 2 weeks for 8 weeks) or due to indirect effects of the GW3965 CM on cell types apart from endothelial cells. Nevertheless, the precise mechanism by which the secretome exerts this effect on the endothelium to mitigate lesion development is yet to be identified. Signals transduced from the secretome are likely derived from proteins^24^, ^52^, ^53^, ^54^, ^55^, ^56^, ^57^ or components of extracellular vesicles (ie, miRNAs).^58^, ^59^, ^60^ As such, investigation and fractionation of the LXR‐dependent changes to the EOC secretome is warranted to identify potential effector pathways of endothelial cells that lead to decreased monocyte adhesion, including unbiased proteomic approaches and analysis of RNA species contained within the ligand‐treated EOC secretome. A preliminary proteomic analysis of unfractionated day 7 EOC secretomes found elevated levels of several known LXR targets in the CM from GW3965‐treated vs vehicle‐treated cells. For example, phospholipid transfer protein (PLTP), apoptotic inhibitory factor AIM/CD5L, IL18 binding protein (IL18BP) and lipoprotein lipase (LPL) were increased over 2‐fold. Intriguingly, among these secreted proteins, PLTP, CD5L, and LPL have been reported to exacerbate or be associated with advanced atherosclerosis,^61^, ^62^, ^63^, ^64^ whereas IL18BP is anti‐atherogenic.^65^ Further experiments are needed to fractionate and assess all candidates alone or in combination to determine how, if any, exert their protective effect in the CM. Nevertheless, we show here that GW3965‐treatment of EOCs derived from patients with CAD produces a secretome that also reduces monocyte‐endothelial adhesion (Figure 2B), indicating that LXR agonist treatment to EOCs could potentially be a translatable therapeutic for patients with cardiovascular disease.

The administration of EOCs to atherosclerotic mouse models has been previously shown to promote plaque stabilization and decrease plaque formation, primarily through endothelial engraftment.^66^, ^67^, ^68^, ^69^ However, those studies were all performed in mouse models of plaque regression that targeted the later stages of atherosclerosis. EOC intervention at the early stages of atherosclerosis, as described herein, has not, to our knowledge, been previously explored. As a complement to our in vitro monocyte‐endothelial adhesion assay, we evaluated the therapeutic potential of LXR treatment to EOCs in a mouse model of early atherosclerosis. In line with numerous studies and clinical trials (reviewed by Mindur and Swirski^70^) that show the potency of circulating growth factors and cytokines in promoting and/or limiting the development of atherosclerosis, administration of the secreted factors from GW3965‐treated EOCs significantly reduced aortic lesion development in recipient *Ldlr*−/− mice. Of note, the effect of injecting GW3965‐treated EOCs vs GW3965‐treated CM on lesion formation was very similar (44% vs 36%, respectively; Figures 3C and 4C) indicating that the majority of the beneficial effect is derived from the secretome. These data are in line with recent reports in which the EOC secretome has been packaged in nanoparticles or hydrogels to treat acute ischemic diseases.^71^, ^72^

Previous studies determined that in the absence of the cholesterol efflux transporters (*Abca1* and *Abcg1*) in myeloid cells of the bone marrow was insufficient to explain the anti‐atherogenic effects of LXRs described in whole bone marrow transplant experiments.^9^, ^10^, ^11^, ^12^, ^13^, ^14^ Our data support the role of another effector cell (EPC) in contributing to the anti‐atherogenic effects of LXR. Thus, these studies enhance our understanding of LXR cell targets in the bone marrow and provide a unique mechanism to inhibit the development of atherosclerosis. A major drawback to the in vivo use of LXR agonists is the development of hypertriglyceridemia and hepatosteatosis via upregulation of the LXR target gene and lipogenic factor *Srebp1c*, in the liver.^73^, ^74^, ^75^ Our data demonstrate that the cell‐free secretome of LXR‐treated EOCs is sufficient to protect against endothelial damage and early atherosclerosis. These data therefore support a role for ex vivo treatment of patient‐derived EOCs as an avenue of intervention that would elicit the anti‐atherogenic effects of LXRs in the EOCs without the hepatosteatosis associated with systemic LXR agonism.

## 5 CONCLUSION

Our data demonstrate the potential of ex vivo treatment of patient‐derived EOCs with LXR agonists as a novel therapy to decrease the endothelial defects associated with atherosclerosis and reduce plaque burden during its progression. Further experiments will need to be performed to examine the long‐term efficacy and any potential toxicity that may be associated with administering CM prior to human studies.

## CONFLICT OF INTEREST

The authors declared no potential conflicts of interest.

## AUTHOR CONTRIBUTIONS

A.R.: conception and design, collection and/or assembly of data, data analysis and interpretation, manuscript writing, final approval of manuscript; S.A.S.: collection and/or assembly of data, data analysis and interpretation, final approval of manuscript; R.T., M.F.S.: collection and/or assembly of data, final approval of manuscript; R.G.J., T.S., B.H.: provision of study material or patients, collection and/or assembly of data, final approval of manuscript; K.J.R.: data analysis and interpretation, final approval of manuscript; C.L.C.: conception and design, financial support, data analysis and interpretation, manuscript writing, final approval of manuscript.

## Supporting information


**Supporting Information Figure S1** Differentiation of EOCs in the presence of GW3965 generates a distinct population of EOCs that does not induce myeloid or mesenchymal markers. (A‐C): EOCs from WT and *Lxrαβ*−/− mice were treated with GW3965 and gene expression was performed for (A), *Cd11b*, (B), *Tgfβ*, and (C), *Fsp1*. n = 6 per group. (D‐E): Shotgun proteomic analysis of cell pellets at day 1 or day 9, differentiated in the presence of Veh or GW3965. (D) Protein quantitation of the endothelial marker von Willebrand factor (vWF) using label free quantitation (LFQ). (E) Unsupervised clustering of proteomic data (using LFQ values) demonstrating distinct sub‐populations of EOCs differentiated with Veh vs GW3965. n = 2–3 per group. Data represent the mean ± SEM. *P < 0.05, **P < 0.01, ****P < 0.0001.
**Supporting Information Figure S2.** Incubation with the secretome from GW3965‐treated EOCs does not reduce the expression of adhesion molecules and selectins on activated endothelial cells and is effective at decreasing monocyte‐adhesion when incubated either pre‐ or during TNFα. (A‐C) HUVECs were incubated with conditioned media from treated EOCs and gene expression was assessed for (A) *VCAM1*, (B) *ICAM1*, and (C) *SELE*. n = 6 per group. (D) CM from GW3965‐treated EOCs was applied to HUVECs for either i) 20 hours before TNFα addition, ii) 4 hours concurrent with TNFα addition, or iii) during both periods and the effect on monocyte‐endothelial binding was quantified. Scale bar = 400 μm. n = 3‐6 per group. Data represent the mean ± SEM. *P < 0.05, **P < 0.01, ****P < 0.0001.
**Supporting Information Figure S3.** Injection of CM from vehicle or GW3965 treated EOCs does not impact the systemic inflammatory markers (A) plasma IL‐6 levels or (B) spleen weight to body weight ratio. n = 11‐12 per group. Data represent the mean ± SEM.
**Supporting Information Table S1.** List of mouse primers.
**Supporting Information Table S2.** List of human primers.
**Supporting Information Table S3.** Circulating immune cells in splenectomized *Ldlr*‐knockout mice receiving EOCs.
**Supporting Information Table S4.** Circulating immune cells in *Ldlr*‐knockout mice receiving conditioned media derived from treated EOCs

## Data Availability

The data that support the findings of this study are available on request from the corresponding author. The mass spectrometry proteomics data have been deposited to the ProteomeXchange Consortium (http://proteomecentral.proteomexchange.org) via the PRIDE partner repository^76^ with the dataset identifier PXD021993.
