# Supplementary material for: The secretome of liver X receptor agonist‐treated early outgrowth cells decreases atherosclerosis in Ldlr−/− mice
Source: Stem Cells Transl Med. 2020 Nov 24;10(3):479–91. doi: 10.1002/sctm.19-0390 (PMC7900590; doi:10.1002/sctm.19-0390)
Supplement: Supplementary file 1 — Supporting Information Figure S1 Differentiation of EOCs in the presence of GW3965 generates a distinct population of EOCs that does not induce myeloid or mesenchymal markers. (A‐C): EOCs from WT and Lxrαβ−/− mice were treated with GW3965 and gene expression was performed for (A), Cd11b, (B), Tgfβ, and (C), Fsp1. n = 6 per group. (D‐E): Shotgun proteomic analysis of cell pellets at day 1 or day 9, differentiated in the presence of Veh or GW3965. (D) Protein quantitation of the endothelial marker von Willebrand factor (vWF) using label free quantitation (LFQ). (E) Unsupervised clustering of proteomic data (using LFQ values) demonstrating distinct sub‐populations of EOCs differentiated with Veh vs GW3965. n = 2–3 per group. Data represent the mean ± SEM. *P < 0.05, **P < 0.01, ****P < 0.0001. Supporting Information Figure S2. Incubation with the secretome from GW3965‐treated EOCs does not reduce the expression of adhesion molecules and selectins on activated endothelial cells and is effective at decreasing monocyte‐adhesion when incubated either pre‐ or during TNFα. (A‐C) HUVECs were incubated with conditioned media from treated EOCs and gene expression was assessed for (A) VCAM1, (B) ICAM1, and (C) SELE. n = 6 per group. (D) CM from GW3965‐treated EOCs was applied to HUVECs for either i) 20 hours before TNFα addition, ii) 4 hours concurrent with TNFα addition, or iii) during both periods and the effect on monocyte‐endothelial binding was quantified. Scale bar = 400 μm. n = 3‐6 per group. Data represent the mean ± SEM. *P < 0.05, **P < 0.01, ****P < 0.0001. Supporting Information Figure S3. Injection of CM from vehicle or GW3965 treated EOCs does not impact the systemic inflammatory markers (A) plasma IL‐6 levels or (B) spleen weight to body weight ratio. n = 11‐12 per group. Data represent the mean ± SEM. Supporting Information Table S1. List of mouse primers. Supporting Information Table S2. List of human primers. Supporting Information Table S3. Circulating immune [file SCT3-10-479-s001.docx]

**Supporting Information Figures and Tables**

**

**

**Supporting Information Figure S1.** Differentiation of EOCs in the presence of GW3965 generates a distinct population of EOCs that does not induce myeloid or mesenchymal markers. **(A-C):** EOCs from WT and *Lxrαβ*-/- mice were treated with GW3965 and gene expression was performed for **(A)**, *Cd11b*, **(B)**, *Tgfβ*, and **(C)**, *Fsp1*. n=6 per group. **(D-E):** Shotgun proteomic analysis of cell pellets at day 1 or day 9, differentiated in the presence of Veh or GW3965. **(D)** Protein quantitation of the endothelial marker von Willebrand factor (vWF) using label free quantitation (LFQ). **(E)** Unsupervised clustering of proteomic data (using LFQ values) demonstrating distinct sub-populations of EOCs differentiated with Veh vs. GW3965. n=2-3 per group. Data represent the mean ± SEM. **P*<0.05, ***P*<0.01, *****P*<0.0001.

**
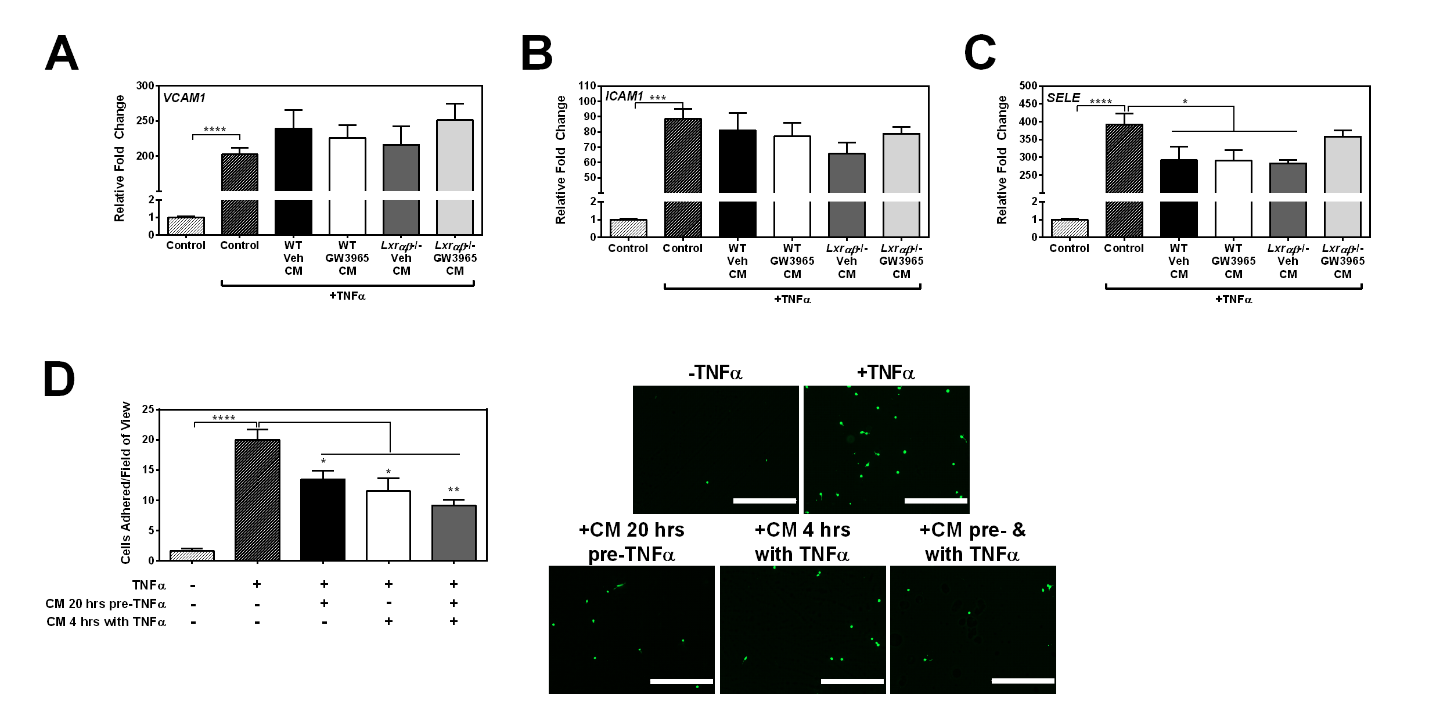
**

**Supporting Information Figure S2.** Incubation with the secretome from GW3965-treated EOCs does not reduce the expression of adhesion molecules and selectins on activated endothelial cells and is effective at decreasing monocyte-adhesion when incubated either pre- or during TNFα. **(A-C)** HUVECs were incubated with conditioned media from treated EOCs and gene expression was assessed for **(A)** *VCAM1*, **(B)** *ICAM1*, and **(C)** *SELE*. n=6 per group. **(D)** CM from GW3965-treated EOCs was applied to HUVECs for either i) 20 hrs before TNFα addition, ii) 4 hrs concurrent with TNFα addition, or iii) during both periods and the effect on monocyte-endothelial binding was quantified. Scale bar = 400 μm. n=3-6 per group. Data represent the mean ± SEM. **P*<0.05, ***P*<0.01, *****P*<0.0001.





**Supporting Information Figure S3.** Injection of CM from vehicle or GW3965 treated EOCs does not impact the systemic inflammatory markers **(A)** plasma IL-6 levels or **(B)** spleen weight to body weight ratio. n=11-12 per group. Data represent the mean ± SEM.

**Supporting Information Table S1.** List of mouse primers.

| **Gene (abbrev.)** | **Accession number** | **Forward Primer (5’ 🡪 3’)** | **Reverse Primer (5’ 🡪 3’)** |
| --- | --- | --- | --- |
| Fibroblast specific protein 1 (*Fsp1*) | NM_011311.2 | aagctgaacaagacagagctcaag | atgaagctgcattccagaaggt |
| Integrin αM (*Cd11b*) | NM_0010829601.1 | CAGCACAAGCCGGTGTCA | GACATATTCACAGCCTCTGGAGGTA |
| Kinase domain receptor (*Vegfr2*) | NM_010612.2 | GATGCAGGAAACTACACGGTCAT | AGGCGAGATCAAGGCTTTCTC |
| Peptidylprolyl isomerase A (*Cyclophilin*) | NM_011149.2 | CAACGATAAGAAGAAGGGACCTAAA | CGTCCTACAGATTCATCTCCAATTT |
| Protein tyrosine phosphatase, receptor type, C (*Cd45*) | NM_011210.3 | AAGCACTGACCCTCCAAGCA | CATGGCAGCACATGTTTGC |
| Transforming growth factor β (*Tgfβ*) | NM_011577.1 | gcagtggctgaaccaagga | agcagtgagcgctgaatcg |
| VE-cadherin (*Cd144*) | NM_009868.4 | TGGCCAAAGACCCTGACAA | ACTGGTCTTGCGGATGGAGTA |

**Supporting Information Table S2.** List of human primers.

| **Gene name (abbrev.)** | **Accession number** | **Forward Primer (5’ 🡪 3’)** | **Reverse Primer (5’ 🡪 3’)** |
| --- | --- | --- | --- |
| Endothelial-leukocyte adhesion molecule (*SELE*) | NM_000450.2 | GGCCACGGTGAATGTGTAGAG | CCCAGTGGGTGACTGCAAAC |
| Intercellular cell adhesion molecule (*ICAM1*) | NM_000201.2 | GCCGGCCAGCTTATACACAA | TGGCCACGTCCAGTTTCC |
| Peptidylprolyl isomerase A (*CYCLOPHILIN*) | NM_000942.4 | ggagatggcacaggaggaa | gcccgtagtgcttcagttt |
| Vascular cell adhesion molecule 1 (*VCAM1*) | NM_001078.3 | AAACAAAGGCAGAGTACGCAAAC | GGCTGACCAAGACGGTTGTATC |

**Supporting Information Table S3.** Circulating immune cells in splenectomized *Ldlr*-knockout mice receiving EOCs.

|  | Saline | Veh EOC | GW3965 EOC |
| --- | --- | --- | --- |
| RBC (10^12^ cells/L) | 10.0 ± 0.3 | 9.9 ± 0.2 | 10.4 ± 0.1 |
| WBC (10^9^ cells/L) | 13 ± 1 | 12 ± 1 | 13.0 ± 0.8 |
| Lymphocyte (10^9^ cells/L) | 10 ± 3 | 10 ± 1 | 10.1 ± 0.6 |
| Platelet (10^9^ cells/L) | 590 ± 40 | 670 ± 20 | 610 ± 30 |
| Monocyte (10^9^ cells/L) | 0.55 ± 0.06 | 0.59 ± 0.05 | 0.59 ± 0.05 |
| Neutrophil (10^9^ cells/L) | 2.7 ± 0.4 | 2.1 ± 0.1 | 2.2 ± 0.4 |

n=10-11 mice per group. Data represent the mean ± SEM. RBC: red blood cell, WBC: white blood cell.

**Supporting Information Table S4.** Circulating immune cells in *Ldlr*-knockout mice receiving conditioned media derived from treated EOCs.

|  | Control | Veh CM | GW3965 CM |
| --- | --- | --- | --- |
| RBC (10^12^ cells/L) | 10.0 ± 0.2 | 10.2 ± 0.1 | 10.3 ± 0.1 |
| WBC (10^9^ cells/L) | 6.7 ± 0.5 | 8.3 ± 0.5 | 8.0 ± 0.5 |
| Lymphocyte (10^9^ cells/L) | 5.2 ± 0.4 | 6.3 ± 0.3 | 6.2 ± 0.4 |
| Platelet (10^9^ cells/L) | 580 ± 50 | 670 ± 30 | 650 ± 30 |
| Monocyte (10^9^ cells/L) | 0.28 ± 0.03 | 0.41 ± 0.04 | 0.44 ± 0.04* |
| Neutrophil (10^9^ cells/L) | 1.3 ± 0.1 | 1.5 ± 0.2 | 1.4 ± 0.2 |

n=11-12 mice per group. Data represent the mean ± SEM. **P*<0.05 compared to Control. RBC: red blood cell, WBC: white blood cell.
